# High detection rate of circulating-tumor DNA from cerebrospinal fluid of children with central nervous system germ cell tumors

**DOI:** 10.1186/s40478-024-01886-w

**Published:** 2024-11-20

**Authors:** Yoshiko Nakano, Ian Burns, Liana Nobre, Robert Siddaway, Mansuba Rana, Cody Nesvick, Andrew Bondoc, Michelle Ku, Richard Yuditskiy, Dennis T. L. Ku, Matthew M. K. Shing, Kevin K. F. Cheng, Ho-Keung Ng, Anirban Das, Julie Bennett, Vijay Ramaswamy, Annie Huang, David Malkin, Birgit Ertl-Wagner, Peter Dirks, Eric Bouffet, Ute Bartels, Uri Tabori, Cynthia Hawkins, Anthony P. Y. Liu

**Affiliations:** 1 Division of Haematology/Oncology, The Hospital for Sick Children, 555 University Avenue, Toronto, ON M5G 1X8 Canada; 2 Department of Paediatrics, University of Toronto, Toronto, ON Canada; 3 Department of Paediatrics, The Hospital for Sick Children, Toronto, ON Canada; 4 Department of Paediatrics, University of Alberta, Edmonton, AB Canada; 5 Department of Pediatric Laboratory Medicine, The Hospital for Sick Children, 555 University Avenue, Toronto, ON M5G 1X8 Canada; 6 The Arthur and Sonia Labatt Brain Tumour Research Centre, The Hospital for Sick Children, Toronto, ON Canada; 7 Department of Neurosurgery, The Hospital for Sick Children, Toronto, ON Canada; 8 Department of Paediatrics and Adolescent Medicine, Hong Kong Children’s Hospital, Kowloon, Hong Kong China; 9 Department of Neurosurgery, Hong Kong Children’s Hospital, Kowloon, Hong Kong China; 10 Department of Anatomical and Cellular Pathology, Chinese University of Hong Kong, Shatin, Hong Kong China; 11 Division of Medical Oncology and Hematology, Princess Margaret Cancer Centre, Toronto, ON Canada; 12 Department of Diagnostic and Interventional Radiology, The Hospital for Sick Children, Toronto, ON Canada; 13 Department of Medical Imaging, University of Toronto, Toronto, ON Canada; 14 Department of Laboratory Medicine and Pathobiology, University of Toronto, Toronto, ON Canada; 15 Department of Paediatrics and Adolescent Medicine, School of Clinical Medicine, The University of Hong Kong, Pokfulam, Hong Kong China

**Keywords:** Central nervous system germ cell tumors, Cell-free DNA, Circulating-tumor DNA, Liquid biopsies, Measurable residual disease

## Abstract

Central nervous system germ cell tumors (CNS-GCT) are malignant neoplasms that arise predominantly during adolescence and young adulthood. These tumors are typically sensitive to treatment, but resulting long-term health deficits are common. Additional clinical challenges include surgical risks associated with tumor biopsy, and need to determine treatment response for adapting radiotherapy protocols. The aim of this study was to establish the detectability of circulating-tumor DNA (ctDNA) from cerebrospinal fluid (CSF) of children with CNS-GCT as a potential biomarker. We obtained CSF from patients with CNS-GCT by lumbar puncture or intra-operatively. Cell-free DNA (cfDNA) was extracted and subjected to low-pass whole genome sequencing (LP-WGS). Copy-number alterations (CNAs) were inferred and served as a marker of measurable residual disease (MRD). Comparisons with imaging findings and tumor marker levels were made. A total of 29 CSF samples from 21 patients (16 with germinoma, 5 with non-germinomatous GCT) were sequenced. Twenty samples from 19 patients were collected at diagnosis, and 9 samples from 7 patients were collected during or after therapy. Among the diagnostic samples, CNAs were detected in samples from 17/19 patients (89%), which included 8 with marker-negative tumors. Specific clinical scenarios suggested that serial cfDNA analysis may carry utility in tracking treatment responses as well as clarifying indeterminate imaging findings. Our results provide evidence for the high-sensitivity in detecting ctDNA from CSF of CNS-GCT patients using LP-WGS, with potential utility for non-invasive diagnosis and disease monitoring in upcoming CNS-GCT studies.

**Supplementary Information:**

The online version contains supplementary material available at 10.1186/s40478-024-01886-w.

## Introduction

Central nervous system germ cell tumors (CNS-GCTs) are neoplasms that predominantly arise during adolescence and young adulthood [1]. This heterogeneous group of tumors has a higher incidence in Asia and represents one of the most common types of pediatric CNS tumor in the region [2–4]. CNS-GCTs are divided into germinomas and non-germinomatous GCTs (NGGCTs); common anatomical sites include the pineal gland, sellar-suprasellar region, and basal ganglia. The diagnosis of CNS-GCT relies on a combination of imaging, biochemical markers, and histology especially for non-secreting lesions. Measuring levels of alpha-fetoprotein (AFP) and beta human chorionic gonadotropin (β-hCG) in cerebrospinal fluid (CSF) and serum are essential components of the initial diagnostic workup and subsequent disease surveillance. Markedly elevated AFP and/or β-hCG, along with compatible imaging features, are diagnostic of NGGCTs, whereas isolated, mildly elevated β-hCG may suggest syncytiotrophoblast-containing germinomas. While a chemotherapy-only approach resulted in cure for half of the patients with CNS-GCT [5], with combination of chemotherapy and irradiation, disease-free survival rates exceeded 80–90% [6–9].

Despite such favorable outcomes, challenges in the management of CNS-GCTs remain. Surgical sampling of these deep-seated lesions carries inherent risks, such as further disruption to the hypothalamic-pituitary axis for sellar-suprasellar lesions. Standardized normal cut-offs for AFP and β-hCG, as well as thresholds for delineation between germinomas and NGGCTs, also remain elusive. Moreover, the interpretation of imaging findings, particularly for determining remission status after induction chemotherapy, is often not straightforward, hindering efforts to de-escalate radiotherapy in good responders for mitigation of treatment-associated late effects. To overcome these challenges, we hypothesize that CSF-based liquid biopsy approaches, which have proven feasibility and utility in other pediatric CNS tumors like medulloblastomas, may complement current diagnostic and surveillance strategies in CNS-GCTs [10, 11]. Similar to medulloblastomas, CNS-GCTs are known to harbor frequent copy number alterations (CNAs) that can be inferred by cost-effective low-pass whole-genome sequencing (LP-WGS) and used as surrogate markers of measurable residual disease (MRD) at the molecular level [12–15]. We therefore aimed to assess the feasibility of detecting CSF-derived circulating tumor DNA (ctDNA) in children with CNS-GCTs using LP-WGS.

## Materials and methods

### Patients and sample collection

This study was approved by the Hospital for Sick Children Research Ethical Board (REB # 1000071241) and the Hong Kong Children’s Hospital Research Ethics Committee (HKCH-REC-2020-068). Written informed consent was obtained from all patients, their parents or guardians. CSF samples were collected from consecutive patients with CNS-GCT consenting to the study between December 2020 and June 2024 at the Hospital for Sick Children, Toronto and Hong Kong Children’s Hospital, Hong Kong. CSF samples were obtained by lumbar puncture (LP) or intraoperatively as part of routine clinical care. CSF was collected into plain sterile collection tubes or Streck tubes (Streck, La Vista, Nebraska). Patients were classified as having germinoma or NGGCT based on histology (when available) and tumor markers (all cases). β-hCG greater than or equal to 5 IU/L was considered abnormal, and AFP > 10 ng/mL and/or β-hCG > 100 IU/L/mL as diagnostic of NGGCT, based on criteria by the Children’s Oncology Group [8, 9]. CSF samples collected into plain tubes were processed within 1 h of collection and those collected into Streck tubes were processed within 5 days. CSF was centrifuged at 1000*g* at room temperature for 10 min. The supernatant was stored at − 80 °C until cell-free DNA (cfDNA) extraction. Patients were treated per the Children’s Oncology Group ACNS1123 study with induction chemotherapy and risk-adapted radiation therapy (Hong Kong Children’s Hospital) [8, 9] or institutional modified radiation regimen [16].

### Extraction of cfDNA from CSF samples

cfDNA was extracted using the QIAamp Circulating Nucleic Acid Kit (Qiagen, Hilden, Germany) based on the manufacturer’s protocol. cfDNA was eluted in 60uL of AVE buffer and stored at − 20 °C until library preparation. Total DNA was quantified using Qubit dsDNA Quantification assay (high sensitivity kit, Thermo Fisher, Waltham, MA, USA), whereas cfDNA (75–300 bp) was quantified using Cell-free DNA ScreenTape assay of the TapeStation system (Agilent Technologies, Santa Clara, USA).

### Library preparation and sequencing

A maximum of 30 ng of cfDNA was used as input for library preparation. End repair, A-tailing and ligation of UMI were performed using TruSight Oncology 500 ctDNA v2 (Illumina, San Diego, USA). Ligation of UDI adapter, purification, and target enrichment for panel sequencing were performed using Twist library preparation kits according to the manufacturer's protocols (Twist Bioscience). Libraries were quantified on the 2100 Bioanalyzer system (Agilent Technologies, Santa Clara, USA). Where relevant, hybrid capture was performed using 500 ng library DNA and Twist target enrichment protocol to a 21-gene targeted panel (*BRAF, CDKN2A, EGFR, FGFR1, FGFR2, FGFR3, H3F3A, HIST1H3B, HIST1H3C, IDH1, IDH2, KRAS, MYB, MYL1, MYC, MYCN, PDGFRA, PIK3CA, PTPN11, TERT, TP53*). The panel was synthesized by Twist Bioscience and hybrid capture was performed according to the manufacturer’s recommendation. Samples were sequenced on NextSeq 550 (Illumina) as paired end 150-base pair reads. LP-WGS was performed for all samples, with sequenced coverage from 0.04–3.09× (median 0.34×) achieved. For samples with LP-WGS and targeted panel capture, sequencing was performed separately.

### Bioinformatic analysis

Raw sequencing reads were aligned to human genome build GRCh37 using bwa-mem v0.7.12, and further processed using GATK v4.2.6 according to recommended best practices. Copy number analysis for cfDNA was performed using ichorCNA using default parameters and a bin size of 500 kb [17]. For SNV calling, BaseSpace was used in a dedicated instance located in Canada compliant with relevant data privacy regulations. Analysis was performed with DRAGEN v.3.10.4 and variants were interpreted with BaseSpace variant interpreter v.2.17; variants were annotated with COSMIC and ClinVar databases, while alterations recorded in population databases (i.e., > 0.01 allele frequency in 1000 Genomes, TOPMed, 1000 Genomes project, and NHLBI Exome Project) were excluded.

### Copy number profiling for tumor DNA

DNA was extracted from formalin-fixed paraffin-embedded (FFPE) tumor samples using CELLDATA DNAstorm 2.0 FFPE DNA Extraction kit (Biotium, Fremont, USA). 150 ng of tumor genomic DNA was used for library preparation. After mechanical fragmentation and DNA repair with NEBNext FFPE DNA repair v2 module (New England Biolabs, Massachusetts, USA), libraries were constructed using Twist universal adapter system (Twist Bioscience, California, USA) and NEBNext FFPE DNA repair v2 module (New England Biolabs, Massachusetts, USA). LP-WGS and copy number analysis were performed using the same bioinformatic pipeline (ichorCNA) as described above.

### Statistical analysis

The Mann–Whitney U test was used for comparison between continuous variables. Analysis was performed using GraphPad Prism 10.2.2 (GraphPad Software, Boston, USA).

## Results

### Patient characteristics, CSF samples, and cfDNA extraction

Samples from 21 patients, including 16 male and 5 female were included (Table 1). Sixteen patients were diagnosed with germinoma and 5 with NGGCT; 14 patients were diagnosed by histology, and 7 did not undergo tumor biopsy. Twenty patients were evaluated at primary diagnosis or during upfront therapy, while for one patient the first CSF sample was collected at the time of disease relapse (GCT014). The median age at the time of first CSF collection on study was 13 years (range: 7–17) and to date, all patients recruited upfront remained alive without experiencing any events at last follow-up (1.7–43.9 months from study enrollment). Primary tumor locations included the suprasellar region (N = 7), basal ganglia (N = 4), pineal gland (N = 4), bifocal (N = 3), basal ganglia and suprasellar region (N = 2), and the temporal lobe (N = 1, patient at relapse). Five had radiographic evidence of periventricular metastasis. In total, 29 CSF samples were analyzed. Twenty samples from 19 patients were collected at diagnosis, among these, 12 samples were obtained via LP, 5 intraoperatively, and 3 via external ventricular drain (EVD). Intraoperative samples were collected from the ventricles endoscopically before tumor biopsy (if indicated). Nine samples from seven patients were collected during therapy (N = 8) or after completion of therapy (N = 1); all of these were obtained through LP.

**Table 1:** Clinicopathological characteristics and summary of liquid biopsy

Clinical information							Liquid biopsy (at diagnosis)				Liquid biopsy (follow-up)				
Study ID	Age (y)*	Sex	Diagnosis	Location	Serum β-hCG (IU/L)	CSF β-hCG (IU/L)	CSF (mL)	Collection method	cfDNA/CSF (ng/mL)	CNA	Timing	CSF (mL)	Collection method	cfDNA/CSF (ng/mL)	CNA
GCT001	17	M	Germinoma	BG	12	12	2	LP	0.27	Neg	–	–	–	–	–
GCT002	16	F	Germinoma^¶^	BG	< 1	< 1	0.7	LP	1.41	Pos	–	–	–	–	–
GCT003	12	M	Germinoma^¶^	Sup	< 1	7	2	Op	5.97	Pos	–	–	–	–	–
							3	LP	1.76	Pos	–	–	–	–	–
GCT004	13	M	NGGCT^¶^	Bi	16	107	1.5	EVD	112.8	Pos	–	–	–	–	–
GCT005	12	M	Germinoma	BG	24	57	2	LP	0.2	Neg	Pre-RT	2	LP	0.15	Neg
GCT006	12	M	Germinoma^¶^	Sup	< 1	< 1	1.8	LP	0.21	Pos	–	–	–	–	–
GCT007	14	M	NGGCT	BG	14	148	1	LP	47.22	Pos	–	–	–	–	–
GCT008	8	F	NGGCT^¶^	Sup, Vent	363	124	2.7	Op	0.1	Pos	Pre-RT	2	LP	0.09	Neg
GCT009	17	M	Germinoma^¶^	Bi, Vent	< 1	66	1.5	Op	84.8	Pos	Pre-RT	2	LP	0.21	Pos
											Off Tx	2	LP	0.1	Neg
GCT010	7	M	NGGCT^¶^	BG, Sup, Vent	24^§^	35^§^	2	LP	7.7	Pos	Pre-RT	2	LP	0.06	Neg
GCT011	12	M	Germinoma	Sup	< 1	4	5	LP	0.32	Pos	–	–	–	–	–
GCT012	10	M	Germinoma^¶^	Pineal	< 1	< 1	5	Op	0.39	Pos	–	–	–	–	–
GCT013	17	F	Germinoma^¶^	Sup, Vent	< 1	< 1	5	LP	0.26	Pos	–	–	–	–	–
GCT014	15	M	Germinoma^¶^	Vent, Hemi	< 1	25^§^^§﻿^	6	EVD	23.8	Pos	On CTx	4	LP	0.04	Pos
											Pre-RT	4.5	LP	0.09	Neg
GCT015	15	M	Germinoma	Bi	< 1	38	4.8	LP	0.81	Pos	–	–	–	–	–
GCT016	14	M	Germinoma^¶^	Pineal	< 1	< 1	2	LP	0.2	Pos	–	–	–	–	–
GCT017	8	M	Germinoma^¶^	Pineal	< 1	2	9.4	LP	2.9	Pos	–	–	–	–	–
GCT018	15	M	Germinoma^¶^	Pineal	< 1	< 1	5	Op	21.8	Pos	–	–	–	–	–
GCT019	12	F	NGGCT	Sup	51	481	4.8	Op	18.7	Pos					
GCT020	13	M	Germinoma	BG	12	30	–	–	–	–	Pre-RT	2.2	LP	0.15	Neg
GCT021	10	F	Germinoma^¶^	Sup	< 1	< 1	–	–	–	–	On CTx	2	LP	0.12	Neg

^¶^Pathologically diagnosed. *Age at initial CSF sample was collected. ^§^AFP was positive (serum-AFP 28 ng/mL,CSF-AFP 26 ng/mL). ^§§^CSF AFP was positive (1.67 ng/mL)

NGGCT, non-germinomatous germ cell tumor; BG, basal ganglia; Sup, suprasellar; Bi, bifocal; Vent, ventricular; Hemi, hemisphere; hCG, human chorionic gonadotropin; LP, lumbar puncture; Op, intraoperatively; EVD, external ventricular drain; CNA, copy number alterations; Pos, positive; Neg, negative; Pre-RT, before radiation; Tx, treatment; CTx, chemotherapy; ΩY, years

The median volume of CSF used for cfDNA extraction at diagnosis was 2.35 mL (range: 0.7–9.4) and median cfDNA concentration (cfDNA/CSF) was 1.58 ng/mL (range: 0.1–112.8) (Table 1, Supplementary Fig. 1A). CSF β-hCG was positive (> 5 IU/L) in 12 patients (57%) and AFP was positive in two patients (GCT010 and GCT014). CSF cytology was positive for malignant cells in patient GCT004 and negative in all other patients. Although cfDNA concentrations varied among samples, ventricular samples (i.e., CSF collected intraoperatively or from EVD) had relatively higher concentrations compared to LP samples (mean 33.5 ng/mL vs 5.2 ng/mL) (Supplementary Figure S1).

### ctDNA detection at diagnosis

**Fig. 1:**
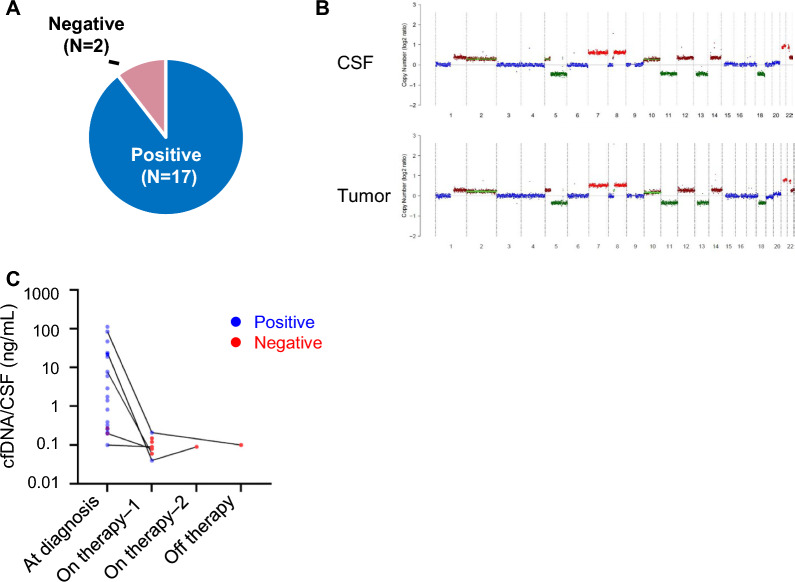
**A** Measurable disease positivity from cerebrospinal fluid (CSF) samples collected at diagnosis or initial staging. **B** Exemplary case (GCT003) depicting tumor-concordant copy-number alterations inferred from low-pass whole-genome sequencing (LP-WGS) of cell-free DNA (cfDNA) extracted from CSF at diagnosis. **C** cfDNA concentrations in CSF samples collected at diagnosis, during therapy and after therapy, demonstrating a decreasing trend with therapy in our cohort

As a surrogate for the presence of molecular disease or ctDNA, chromosomal CNAs were detected in diagnostic CSF samples for 17/19 patients (89%) (Fig. 1A). All 8 patients with negative tumor markers had detectable CNAs (Supplementary Table S1). The 2 CNA-negative samples were both from patients with basal ganglia tumors who had no evidence of pituitary dysfunction (GCT001 and GCT005). This was in contrast to 2 other patients with basal ganglia tumors where CNAs were detected (GCT002 and GCT007), both of whom displayed features of hypopituitarism suggesting more extensive tumor involvement.

Matched tumor samples from 6 patients were available for LP-WGS. CNAs detected from ctDNA were concordant with those in the matched tumor samples (Fig. 1B and Supplementary Fig. 2). In one patient (GCT003), CSF samples were collected both intraoperatively and by LP before chemotherapy. While the cfDNA concentration was higher from the intraoperative specimen, the CNA profile was consistent between the 2 samples (Supplementary Figure S3).

### Longitudinal CSF analysis with therapy

The cfDNA concentration of samples collected during therapy or after therapy completion was lower compared to samples at diagnosis, and a decreasing trend of concentration with treatment was observed in patients where serial samples were available (Fig. 1C). Of 8 samples collected during therapy, 6 were negative for MRD and 2 were positive. Magnetic resonance imaging (MRI) scans obtained at corresponding time-points showed complete remission of tumors in all 6 patients where MRD was not detected, while MRI findings were equivocal in the 2 patients with MRD-positivity (described below, Case 2—GCT009 and Case 3—GCT014), supporting the potential utility of ctDNA in clarifying inconclusive radiographic results.

### Illustrative cases

#### Case 1: ctDNA positivity predates biochemical diagnosis

**Fig. 2:**
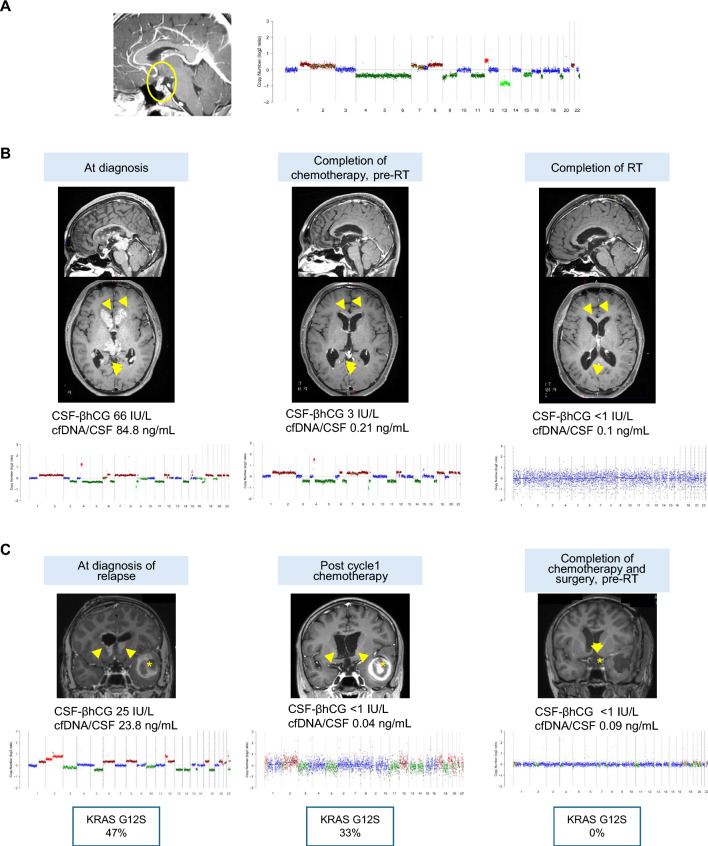
**A** Circulating-tumor DNA (ctDNA) was detected 6 months prior to the borderline elevation of cerebrospinal fluid (CSF) β-hCG in a patient (GCT011) with pituitary stalk thickening and presumed germinoma, offering the potential for early non-invasive diagnostics. **B** Liquid biopsy finding mirrors clinical course in a patient (GCT009) with bifocal germinoma where serial CSF samples were available. Presence of ctDNA in CSF sample collected after completion of chemotherapy (middle panel) suggested residual active disease in spite of equivocal enhancing signal from resolving ventricular disease on imaging (yellow arrow heads). **C** Liquid biopsy detected residual disease and clarified ambiguous radiologic findings after 2 cycles of chemotherapy in a patient with relapsed germinoma (GCT014). With the resolution of ventricular disease (yellow arrowhead) and hemorrhaging temporal lobe lesion (asterisk), repeat liquid biopsy on therapy indicated persistence of molecular disease at both copy-number and mutational (*KRAS*) levels (middle panel). The third CSF collected after completion of chemotherapy, including high-dose chemotherapy, and resection of the left temporal lesion showed no measurable residual disease

A 9-year-old male (GCT011, Fig. 2A) was diagnosed with central diabetes insipidus, and MRI demonstrated absence of the intrinsic T1 signal hyperintensity of the posterior pituitary gland (posterior pituitary bright spot). Follow-up imaging demonstrated progressive thickening of the pituitary stalk: maximal anterior–posterior (AP) diameter was 3.4 mm, 4.2 mm and 5.8 mm at the age of 9, 10 and 11 years respectively. Langerhans cell histiocytosis and CNS-GCT were considered as differential diagnoses. Serum β-hCG was < 1 IU/L and AFP was 2 ng/mL (normal range 1–4 IU/L). At the age of 12 years, CSF was collected and banked; for this sample, β-hCG was 4 IU/L, while AFP was below the limit of detection. Follow-up CSF testing 6 months later showed an increase of β-hCG (7 IU/L) and MRI showed a further increase of the pituitary stalk thickness (from to 6.8 mm to 7.3 mm in maximal AP diameter). Thus, the patient was diagnosed with germinoma without biopsy, and chemotherapy was initiated. When the archival CSF was analyzed for ctDNA, CNAs were readily detected, predating the patient’s diagnosis based on biochemical criteria.

#### Case 2: Liquid biopsy reflects clinical course

A 15-year-old male (GCT009) was diagnosed with bifocal germinoma with periventricular involvement. CSF analysis before initiation of chemotherapy showed high β-hCG (66 IU/L) and CNA positivity. After completion of induction chemotherapy and before the start of radiotherapy, a good response was seen on MRI, although residual periventricular enhancing signal changes remained. At that time point, CSF β-hCG was 3 IU/L, and corresponding cfDNA was positive for CNAs, indicating presence of MRD. Repeat liquid biopsy after completion of radiotherapy that followed was negative for MRD and complete resolution of ventricular lesions was noted on MRI (Fig. 2B). In this case, despite the decrease of markers close to normal level and equivocal MRI findings, CNAs continued to be detectable, highlighting the sensitivity of our assay over conventional approaches for MRD monitoring.

#### Case 3: Liquid biopsy clarifies ambiguous radiologic findings

A 15-year-old male (GCT014) presented with worsening headache 17 months after completing therapy for metastatic, bifocal germinoma. MRI showed intraventricular lesions involving the lateral ventricles and a hemorrhagic lesion in the left temporal lobe. A biopsy from the ventricular lesion confirmed germinoma recurrence. CSF obtained via EVD before chemotherapy was used for liquid biopsy and showed CNAs (Fig. 2C). CSF β-hCG and AFP were 25 IU/L and 1.67 ng/mL respectively (collected on day 5 of chemotherapy). On day 14 of chemotherapy, tumor markers had normalized (CSF β-hCG < 1 IU/L and AFP 0.07 ng/mL). MRI after cycle 1 of chemotherapy showed improvement of the ventricular lesions. However, the tumor burden in the region of the hemorrhagic mass lesion was difficult to assess. Repeat liquid biopsy was performed on day 11 of cycle 2 of chemotherapy, which showed a decrease of cfDNA concentration with only 0.15 ng cfDNA available for sample preparation for sequencing. Although the LP-WGS coverage was low (0.07×), CNAs consistent with previously found CNAs were detected. Additionally, as the tumor analysis was positive for *KRAS* G12S, we performed mutation analysis of cfDNA using our institution’s gene panel. *KRAS* G12S was detected in both CSF samples, with a variant allele frequency of 42% at the time of relapse and 33% at follow-up. While it was impossible to determine disease activity during treatment using MRI due to the presence of hemorrhage, the CNAs detected from the follow-up sample clarified this question. Repeat cfDNA profiling after completion of chemotherapy, autologous stem cell transplantation and surgery (pathology indicating hematoma with absence of tumor cells) was negative for MRD.

## Discussion

Notwithstanding the unique challenges in implementing liquid biopsy techniques in patients with primary CNS tumors, there is increasing literature exploring the profiling of cfDNA extracted from CSF in children with CNS malignancies, particularly embryonal and glial tumors. To our knowledge, our study represents the first report on the performance of LP-WGS in the context of patients with CNS-GCTs. The exceptional detection rate of CNAs of at least 89% for baseline samples from our study patients is among the highest reported in pediatric CNS tumor studies [11, 18, 19]. Furthermore, it is significantly higher than the ctDNA-positive rate of 33% in a study of CNS-GCT that used panel sequencing [20]). This is surprising considering that the majority of our patients had localized disease, and many had imaging features indicating low disease burden. Clear tumor-derived molecular signal could be detected in patients even with just pituitary stalk thickening—a common management dilemma [21].

In the two diagnostic CSF samples where CNAs were not detected (GCT001 and GCT005), copy number profiles from matched tumor specimens were unavailable, and whether the results were true-negative, or the limitation of methodology cannot be resolved. Although CNS-GCTs typically harbor CNAs, CNAs are not detected in approximately 10–25% of CNS-GCTs [12, 15]. Interestingly, in both negative cases, the tumor was located in the basal ganglia, while the two positive cases collected from patients with basal ganglia lesion (GCT002 and GCT007) had hypopituitarism which may suggest occult suprasellar germinoma [22, 23]. While confirmatory studies with larger numbers of patients are needed, the sensitivity of liquid biopsy for GCT may differ depending on tumor location as has been reported for other CNS tumors [11, 24]. In the context of medulloblastoma, ctDNA detection is least successful for SHH-activated lesions, which are often epicentered in the cerebellar hemispheres and away from the ventricular system [11]. The high proportion of positive samples at diagnosis in our study may reflect the frequent occurrence of microscopic ventricular seeding of CNS-GCT, enhancing the tumor-CSF interface [25].

Incorporation of cfDNA analysis into the management of patients with suspected CNS-GCTs may help reduce the need for diagnostic neurosurgical procedures, including for scenarios such as pituitary stalk thickening where tissue sampling may induce pituitary function loss and where sampling error may lead to misdiagnosis [21, 26]. In our cohort, CNAs were detected at baseline in all 13 cases that underwent surgical biopsy, including 8 cases that were marker-negative (Supplementary Table S1 and S2). Ten of the 12 samples collected by LP at diagnosis were positive. Although observing characteristic CNAs such as gains of 1q and 12p are not specific enough to diagnose CNS-GCT [14, 15], the detection of multiple chromosomal aberrations narrows the differential diagnosis by excluding the possibility of hypophysitis, histiocytosis and low-grade glioma, which typically have no or few CNAs [27, 28]. As there is a lack of consensus regarding the optimal cut-offs for tumor markers [29, 30], we envision that cfDNA profiling by LP-WGS, complemented with mutation analysis and methylation studies, will facilitate minimally invasive tumor diagnosis and subtyping that is more biologically relevant and objective when compared to current approaches [12].

Liquid biopsy opens a possible alternative avenue for response monitoring for patients with CNS-GCT, as on-/post-treatment neuroimaging frequently reveals residual signal abnormalities which make it difficult to differentiate between treatment-related changes and residual or recurrent tumor [31]. Of note, MRD was detected in follow-up CSF samples for two patients (GCT009 and GCT014) where imaging showed equivocal findings, and β-hCG was decreased to 3 IU/L (GCT009) or normalized (GCT014). In addition, in case GCT011, liquid biopsy was positive 6 months before the patient was clinically diagnosed with germinoma. These findings, while limited by the sample size, suggest that liquid biopsy may be more sensitive and specific in detecting the presence of active tumor than imaging and tumor markers. In current clinical practice, a patient’s response to chemotherapy dictates their radiotherapy regimen and whether second-look surgery is necessary [1]. Furthermore, with the overarching theme by recent and planned trials to further dose de-escalate patients with low-risk tumors (NCT04684368, NCT06368817) [1, 9], incorporating liquid biopsy to assess response should be further evaluated to enhance risk-stratification and optimization of treatment intensity.

CNAs detected from cfDNA were congruent with those detected from matched tumor tissue in our limited cohort, including two patients with multi-focal primaries (GCT013 and GCT014). This is in contrast to the observation in patients with medulloblastoma where divergence in CNAs were common in tumor-ctDNA comparisons [11]. This discrepancy between tumor types may be biologically driven, considering the superior treatment response in CNS-GCT when compared to medulloblastoma. Whether or not this reflects the extent of tumor heterogeneity warrants further and systematic investigation.

Our study is limited by a relatively small sample size, and the restricted number of patients with longitudinal CSF samples profiled. The lack of clinical events in our study did not allow correlation between liquid biopsy findings and patient outcomes. Nonetheless, our data provide evidence for the first time of a high-sensitivity of detecting ctDNA at baseline in patients with CNS-GCT, offering a strong rationale for the assay to be evaluated in an expanded cohort of patients and integrated with upcoming trial designs to verify the longitudinal dynamics of MRD detectability with therapy. As discussed earlier, we acknowledge that copy-number profiles are not specific diagnostic findings, however, they have utility in narrowing the differential at diagnosis and are relevant from a prognostic standpoint [15]. Since the copy-number-based approach only allows a dichotomized read-out, inference of tumor fraction and extending cfDNA analysis to the epigenomic as well as mutational levels may improve diagnostic specificity.

## Conclusions

In summary, we demonstrated for the first-time the high sensitivity of LP-WGS-based cfDNA profiling for CSF samples from patients with CNS-GCT. This offers an opportunity for earlier and less invasive diagnosis of CNS-GCT. MRD-incorporated risk stratification may identify patients who require lower-doses of, or even no radiation therapy. Such clinical utilities should be validated in prospective studies with larger sample sizes using serially collected CSF at predefined time points.

## Supplementary Information

Additional file 1.

## Data Availability

The datasets used and/or analysed during the current study available from the corresponding author on reasonable request.

## Abbreviations

AFP: Alpha-fetoprotein
β-hCG: Beta human chorionic gonadotropin
cfDNA: Cell-free DNA
CNS-GCT: Central nervous system germ cell tumors
CSF: Cerebrospinal fluid
ctDNA: Circulating-tumor DNA
CNA: Copy-number alterations
EVD: External ventricular drain
FFPE: Formalin-fixed paraffin-embedded
LP-WGS: Low-pass whole genome sequencing
LP: Lumbar puncture
MRI: Magnetic resonance imaging
MRD: Measurable residual disease
NGGCT: Non-germinomatous GCT
