# Supplementary material for: High detection rate of circulating-tumor DNA from cerebrospinal fluid of children with central nervous system germ cell tumors
Source: Acta Neuropathol Commun. 2024 Nov 20;12:178. doi: 10.1186/s40478-024-01886-w (PMC11580361; doi:10.1186/s40478-024-01886-w)
Supplement: Supplementary file 1 — Additional file 1. [file 40478_2024_1886_MOESM1_ESM.pdf]

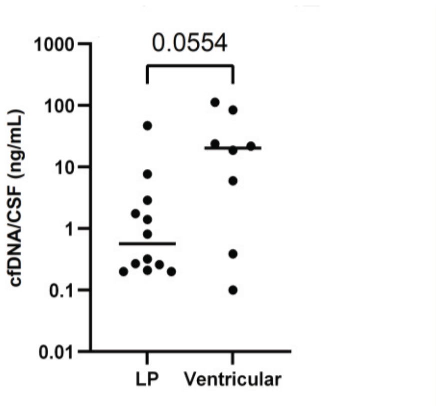

Supplementary Figure S1. cfDNA concentration of lumbar puncture (LP) samples and ventricular samples at diagnosis.

Case No.	CSF	Tumor tissue
GCT003	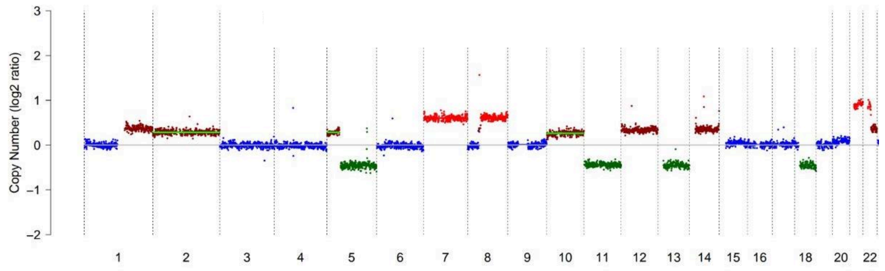	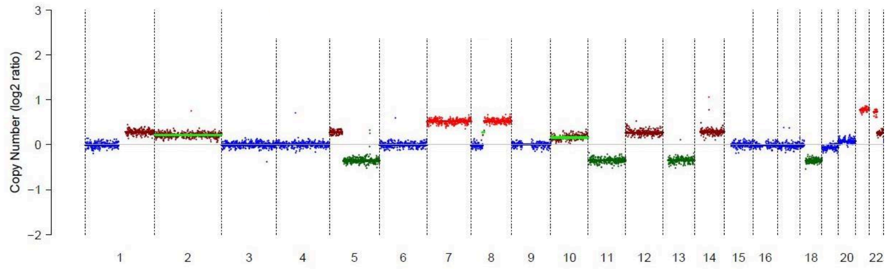
GCT004	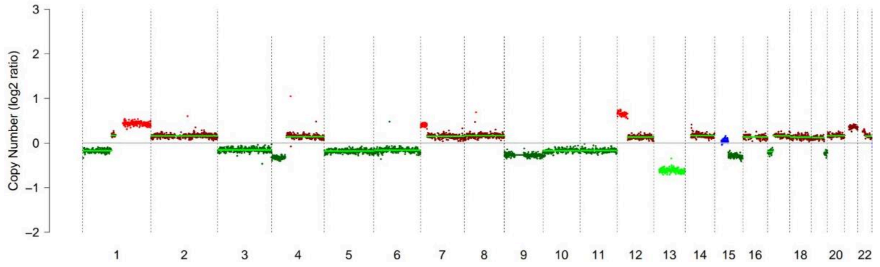	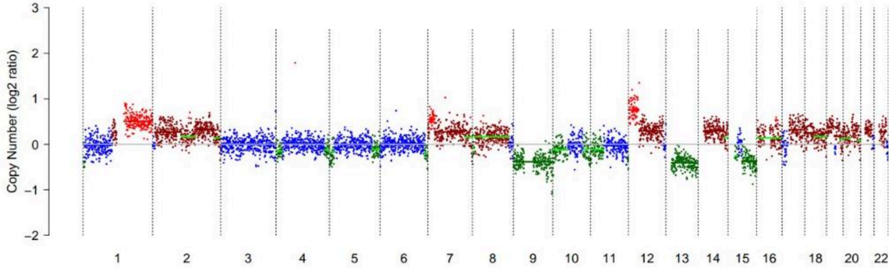
GCT011	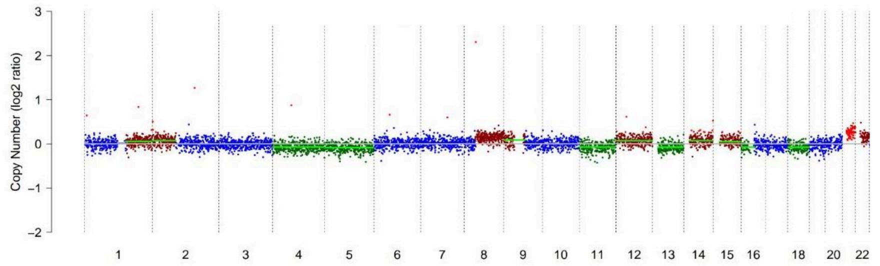	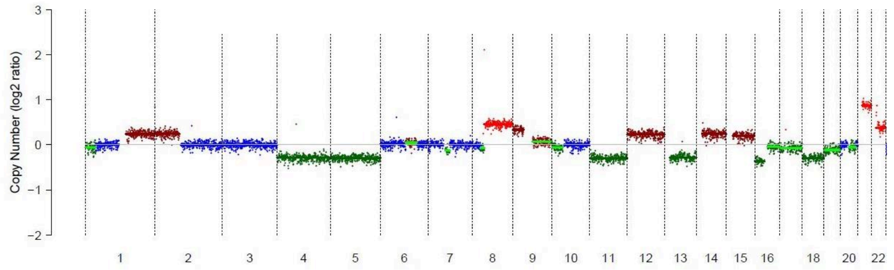
GCT013	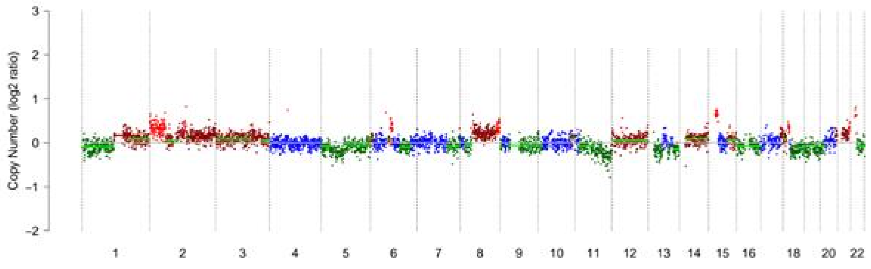	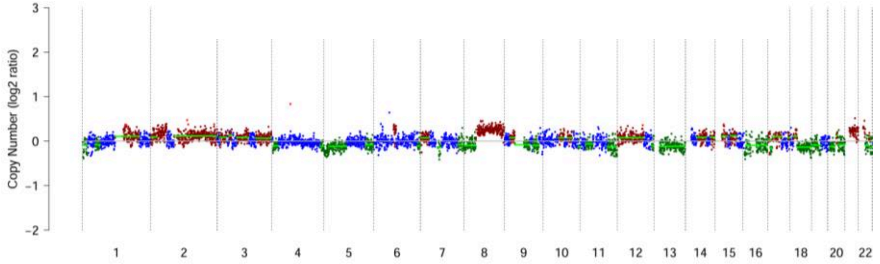

GCT014	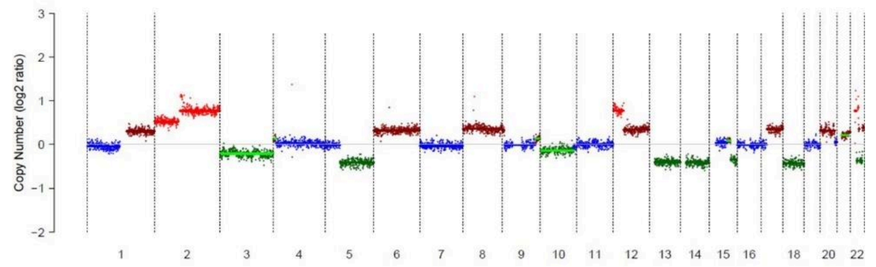	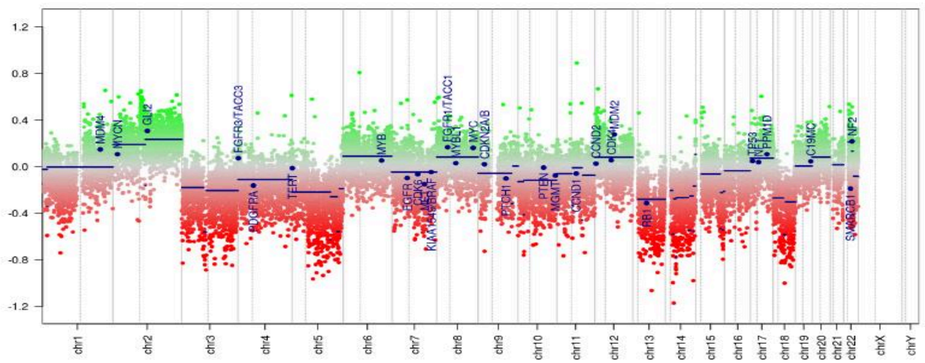
GCT017	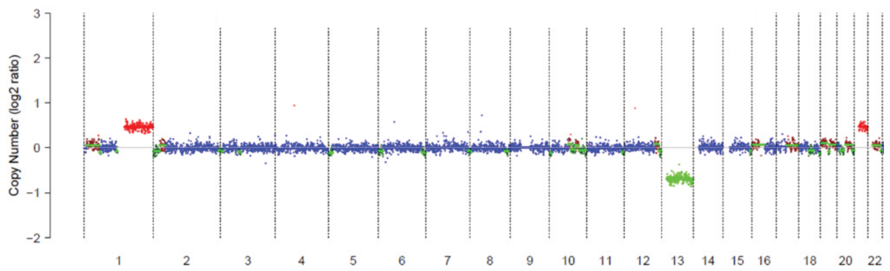	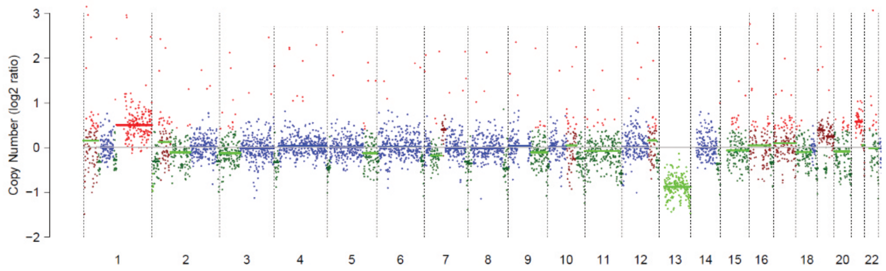

Supplementary Figure S2. Copy number alterations detected in CSF samples and matched-tumor samples.

Intraoperative sample

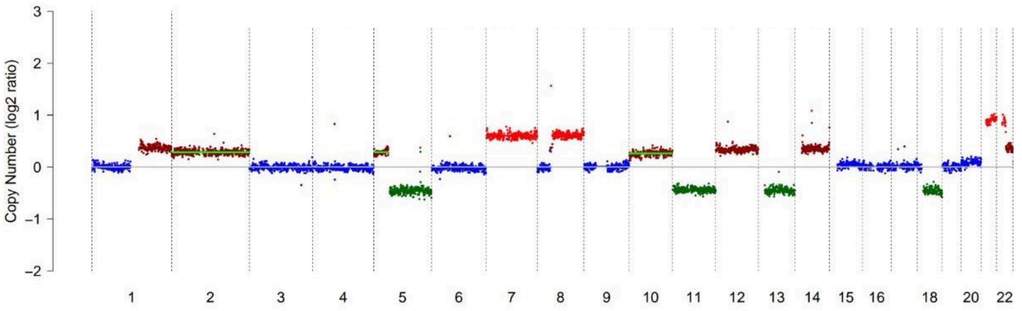

LP sample

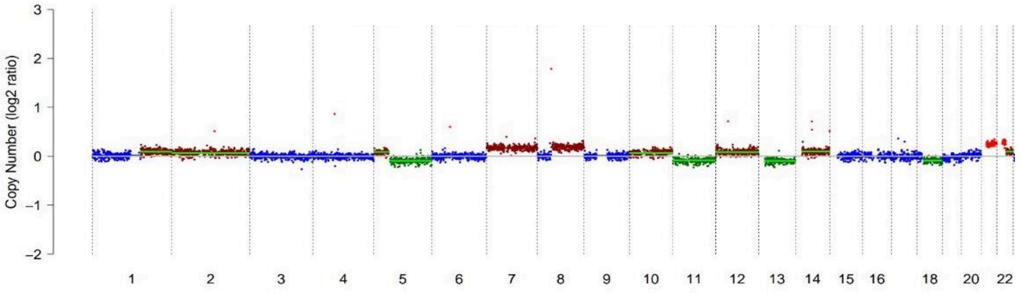

Supplementary Figure S3. Copy number alterations detected in CSF collected intraoperatively and by lumbar puncture (LP) from the same patient (GCT003).

Supplementary Table S1. Comparison of positivity for β-hCG and circulating-tumor DNA (ctDNA) at diagnosis. ctDNA was detected in all marker-negative cases.

		Liquid biopsy (ctDNA)	
		Positive	Negative
CSF β-hCG	Positive	9	2
	Negative	8	0

Supplementary Table S2. Liquid biopsy results in patients with or without tumor biopsy at diagnosis. Circulating-tumor DNA (ctDNA) was detected in all patients who underwent tumor sampling, supporting the potential for less invasive diagnostic strategies.

	Liquid biopsy (ctDNA)	
	Positive	Negative
Not biopsied	4	2
Biopsied	13	0
